# Does a larger estimated fetus compared with prior birthweight increase the risk of cesarean delivery after a prior vaginal birth?

**DOI:** 10.1007/s00404-026-08529-6

**Published:** 2026-07-14

**Authors:** Or Touval, Or Eliner, Sivan Farladansky-Gershnabel, Tal Biron-Shental, Dorit Ravid

**Affiliations:** 1 Department of Obstetrics and Gynecology, Meir Medical Center, Kfar Saba, Israel; 2 Gray Faculty of Medical and Health Sciences, Tel Aviv University, Tel Aviv, Israel

**Keywords:** Cesarean delivery, Estimated fetal weight, Vaginal birth, Interpregnancy weight change, Multiparity

## Abstract

**Purpose:**

To evaluate whether an increase in estimated fetal weight (EFW) relative to the birthweight of a prior vaginal delivery is associated with intrapartum cesarean delivery (CD) or other adverse obstetric outcomes.

**Methods:**

This retrospective cohort study included patients with two consecutive term singleton deliveries (2014–2025). All had a vaginal first delivery and underwent a trial of labor in the second pregnancy. Patients were categorized by the difference between EFW and prior birthweight: higher (> 15%) and similar (-15% to 15%). The primary outcome was intrapartum CD. Multivariable logistic regression adjusted for clinically relevant confounders. Subgroup analysis stratified by EFW (< 3500 vs. ≥ 3500 g) evaluated the effect of absolute fetal size.

**Results:**

The primary analysis included 2849 patients: 2265 in the similar-weight group and 584 in the higher-weight group. The crude intrapartum CD rate was higher in the higher-weight group (5.3% vs 3.0%, *p* = 0.006). After adjustment, weight difference was not independently associated with CD (adjusted odds ratio [aOR] 1.43; 95% CI 0.91–2.27; *p* = 0.12). In subgroup analysis, among patients with EFW ≥ 3500 g, a trend toward increased CD risk was observed in the higher-weight group (aOR 1.68, 95% CI 0.94–2.99, *p* = 0.08).

**Conclusion:**

An increase in EFW relative to prior birthweight was not independently associated with intrapartum CD among patients with a prior vaginal delivery. Among patients with larger absolute EFW, a non-significant trend toward increased intrapartum CD risk was observed, a finding that warrants future investigation. These findings suggest that interpregnancy weight differences alone should not guide delivery planning in otherwise uncomplicated pregnancies.

**Supplementary Information:**

The online version contains supplementary material available at https://doi.org/10.1007/s00404-026-08529-6.

## What does this study adds to the clinical work

**Table:** 

Among patients with a prior vaginal delivery, an increase in estimated fetal weight relative to prior birthweight was not independently associated with intrapartum cesarean delivery. However, among patients with larger absolute estimated fetal size, a non significant trend toward increased cesarean delivery risk was observed, which may warrant further investigation.	

## Introduction

The rate of cesarean delivery (CD) has risen substantially worldwide over recent decades, exceeding 30% in some countries [1, 2]. This trend is concerning, given the well-established association between CD and increased maternal morbidity as well as long-term reproductive and obstetric complications. By contrast, intrapartum primary CD among multiparous patients is relatively uncommon, occurring in approximately 2–5% of cases [3–6].

A prior vaginal delivery is widely considered one of the strongest predictors of subsequent vaginal birth, often reflecting a “proven pelvis”. However, in clinical practice, this reassurance is frequently challenged when the estimated fetal weight (EFW) in a subsequent pregnancy is substantially higher than the birthweight of the previous neonate. In such scenarios, both patients and clinicians may question whether the prior favorable obstetric history remains predictive.

Nevertheless, several risk factors for intrapartum CD in this population have been identified, including obesity, excessive interpregnancy maternal weight gain, advanced maternal age, and various sociodemographic determinants [4, 5, 7, 8].

The effect of increased fetal weight on delivery outcomes is well established. The most notable example is macrosomia, commonly defined as birthweight > 4000 g, which has been associated with adverse maternal and neonatal outcomes, including a higher risk of CD [9, 10].

Importantly, not only the actual birthweight but also the perceived antenatal fetal size may influence delivery outcomes. Prior studies have demonstrated that suspected fetal macrosomia based on EFW, even when the actual birthweight ultimately falls below macrosomia thresholds, is associated with higher intrapartum CD rates. Similar patterns have been observed at EFW below the macrosomia definition [11]. Moreover, the mere presence of an EFW measurement may contribute to higher CD rates [6]. Collectively, these findings suggest that clinicians’ perception of fetal size, rather than birthweight alone, plays an important role in intrapartum decision-making.

Despite this, the clinical implications of relative increase in fetal size between consecutive pregnancies remain unclear. This scenario is frequently encountered in routine obstetric care, where clinicians must counsel multiparous patients whose current fetus appears substantially larger than prior delivery.

Available evidence addressing this clinical scenario is limited and largely extrapolated from studies of trial of labor after cesarean (TOLAC), with inconsistent results [12–15]. Data specifically informing management among patients without a previous uterine scar are notably scarce.

In this study, we aimed to evaluate whether an increased EFW, relative to the birthweight of a prior vaginal birth, is associated with higher risk of CD or other adverse obstetric outcomes. More specifically, we sought to determine whether a prior successful vaginal delivery remains predictive of subsequent delivery outcomes when fetal size is substantially increased. This question is particularly relevant in clinical practice when counseling multiparous patients with a considerable gap between the current EFW and the prior birthweight, especially when weighing decisions regarding induction of labor versus expectant management, as fetal growth progresses.

## Materials and methods

### Study design

This retrospective cohort study included patients with two consecutive term singleton deliveries (37 + 0 to 41 + 6 weeks’ gestation) at a single tertiary medical center between January 2014 and May 2025.

### Study population

Eligible patients had a first vaginal delivery and underwent a trial of labor in their subsequent delivery. Inclusion criteria required both the birthweight in the first delivery and the antepartum sonographic EFW in the index pregnancy to be between 2500 and 4000 g.

Ultrasonographic EFW was obtained within 14 days prior to delivery. All measurements were performed using standard third trimester biometric parameters and were calculated using the Hadlock 4 formula incorporating biparietal diameter (BPD), head circumference (HC), abdominal circumference (AC), and femur length (FL) [16].

Patients with a planned CD in the second delivery were excluded, to allow focused assessment of intrapartum CD risk. Additional exclusion criteria included multiple gestation, known fetal structural or genetic anomalies, preterm (< 37 weeks) or post-term (> 41 + 6) delivery, stillbirth, and missing key obstetric data. Maternal and neonatal characteristics and delivery outcomes were obtained from the electronic medical records system.

The exposure of interest was defined as the relative difference between the antenatal ultrasonographic EFW of the second delivery and the actual birthweight of the first delivery. Patients were categorized based on this difference into two groups: a higher-weight group (EFW > 15% higher than the prior birthweight) and a similar-weight group (-15% to + 15% difference), which served as the reference category. This predefined threshold was selected a priori to exceed the expected variability of sonographic EFW and to identify a substantial difference relative to the previous birthweight, as supported by previous studies and current practice guidelines [17, 18]. Patients with an EFW more than 15% lower than prior delivery birthweight were excluded due to their relatively small number and because this group represents a clinically distinct scenario which is not relevant to the study’s primary question.

### Outcome measures

The primary outcome was intrapartum CD in the second pregnancy. Secondary outcomes included operative vaginal delivery (OVD), maternal complications (postpartum hemorrhage and third- or fourth-degree perineal tears), and neonatal outcomes (shoulder dystocia). All outcomes were assessed in the second delivery only. Data from the first delivery were used solely to derive the change in EFW exposure variable.

### Statistical analysis

Continuous variables are presented as means ± standard deviations (SD) or as median with interquartile ranges (IQR), as appropriate, and were compared using the Student’s *t* test or Mann–Whitney *U* test. Categorical variables are presented as frequencies and percentages and were compared using the Chi-square test or Fisher’s exact test, as appropriate.

Multivariable logistic regression was performed to identify independent associations between clinical and obstetric variables and intrapartum CD. The similar-weight group served as the reference category. Covariates were selected based on clinical relevance and prior literature, and included gestational age at delivery, diabetes status, induction of labor, maternal BMI, height, antepartum sonographic EFW (per 100 g), and previous operative vaginal delivery (OVD).

Missing BMI and height values were handled using multiple imputation by chained equations (MICE). Twenty imputed datasets were generated, incorporating the study exposure, primary outcome, and all covariates included in the multivariable model. Convergence was assessed by evaluating the stability of the imputed BMI distributions and adjusted effect estimates across imputations. Because the missing-at-random (MAR) assumption cannot be formally verified from the observed data alone, complete-case analyses and additional missing-data sensitivity analyses under alternative missing-not-at-random (MNAR) scenarios were performed to evaluate the robustness of the findings.

Additional sensitivity analyses included models incorporating prior birthweight as an independent covariate and temporal analyses adjusting for calendar year. In addition, subgroup analyses stratified by labor onset and the absolute EFW in the index pregnancy were performed. The latter was conducted to evaluate the effect of absolute estimated fetal size, independent of the relative increase from the prior delivery. A threshold of 3500 g was selected a priori based on prior evidence demonstrating an increased risk of intrapartum CD at this level, even below the conventional definition of macrosomia [11].

An additional sensitivity analysis was performed by incorporating patients with an EFW > 15% lower than the previous birthweight into the reference category to assess whether exclusion of this subgroup materially influenced the primary findings.

Adjusted odds ratios (OR) with 95% confidence intervals (CI) are reported. *P* values < 0.05 were considered statistically significant. Statistical analyses were performed using Python (version 3.13) with the pandas, NumPy, SciPy, and statsmodels libraries.

## Results

**Fig. 1:**
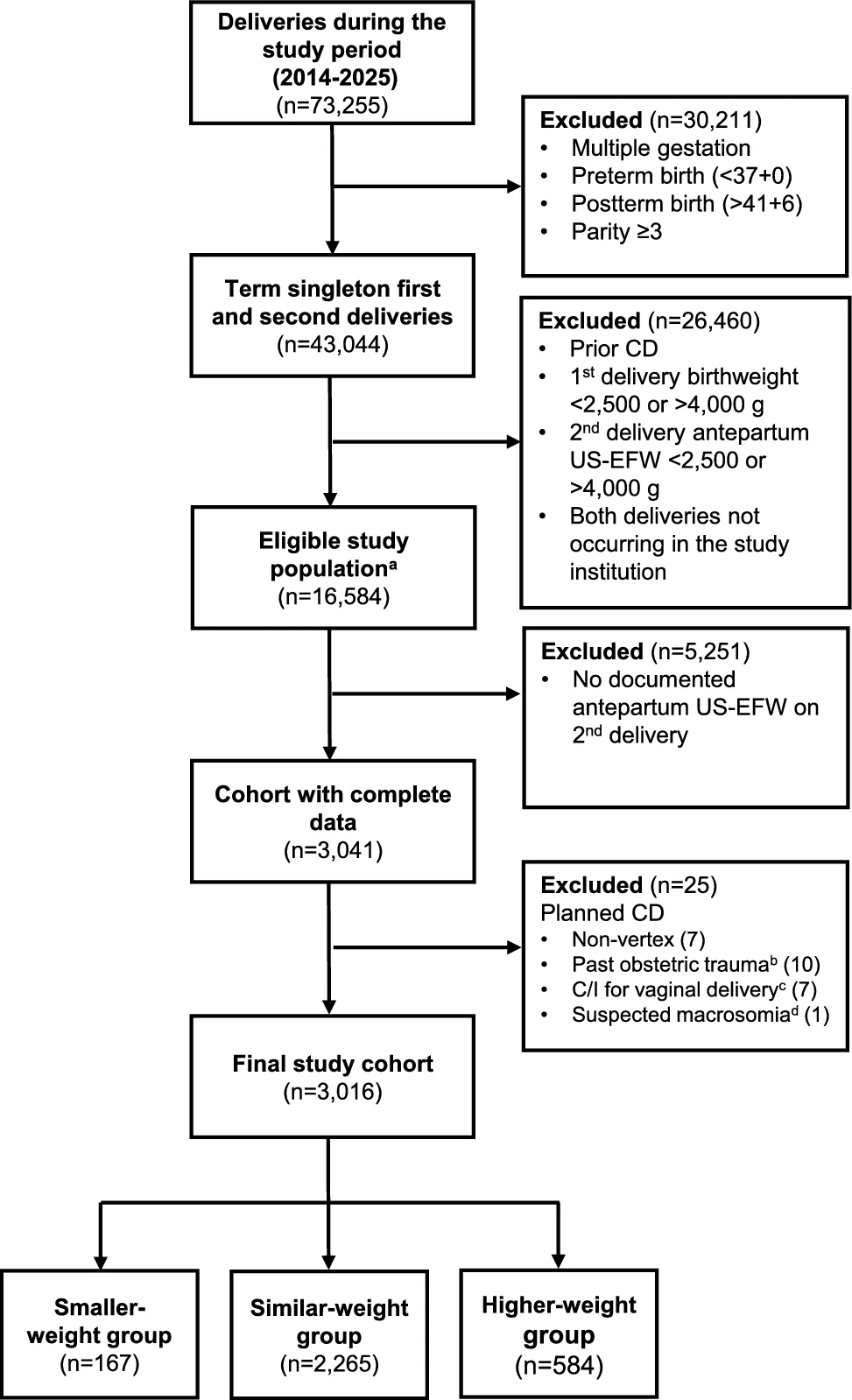
Flow diagram of the study population

During the study period, 73,255 deliveries occurred at our institution. A total of 3,016 patients, representing 6,032 deliveries, met the inclusion criteria and underwent a trial of labor in the second pregnancy. Of these, 2,265 were classified into the similar-weight group, and 584 into the higher-weight group. Additional 167 patients with an EFW more than 15% lower than their prior birthweight were excluded from the primary analysis (Fig. 1).

As expected by the study design, the higher-weight group had a greater mean sonographic EFW in the index pregnancy compared with the similar-weight group (3,496 ± 312 g vs 3,315 ± 330 g, *p* < 0.001). In contrast, birthweight in the prior delivery was markedly lower in the higher-weight group compared with the similar-weight group (2,818 ± 303 g vs 3,299 ± 359 g, *p* < 0.001). Accordingly, the interpregnancy difference in EFW compared with prior delivery birthweight differed substantially between the groups, with a median difference of 626 g (IQR 529 to 781) in the higher-weight group compared with 35 g (IQR -168 to 216) in the similar-weight group (*p* < 0.001). Although several baseline characteristics differed statistically between groups, these differences were small in magnitude (Table 1).

**Table 1:** Maternal demographics and clinical characteristics by change between estimated fetal weight and prior delivery birthweight

Variable	Similar-weight group (*n* = 2,265)	Higher-weight group (*n* = 584)	*p value*
Age (years)	30.39 ± 4.60	31.29 ± 4.52	** < 0.001**
Pregnancy age (weeks)	39.54 ± 0.98	39.61 ± 0.92	0.13
BMI (kg/m^2^)	24.86 ± 5.12 (*n* = 1,219)	25.13 ± 5.22 (*n* = 334)	0.40
Height (cm)	164 ± 6 (n = 1,825)	163 ± 6 (n = 474)	** < 0.001**
Interpregnancy interval (months)	22.5 ± 14.8	24.23 ± 16.3	**0.02**
Hypertensive disorders^a^	44 (1.9%)	18 (3.1%)	0.09
Diabetes^b^	319 (14.1%)	100 (17.1%)	0.06
Sonographic EFW (g)	3,315 ± 330	3,496 ± 312	** < 0.001**
Clinical EFW (g)	3,355 ± 375 (*n* = 1,456)	3,464 ± 316 (*n* = 394)	** < 0.001**
First delivery birthweight (g)	3,299 ± 359	2,818 ± 303	** < 0.001**
Interpregnancy difference in fetal weight^c^ (g)	35 (−168 to 216)	626 (529 to 781)	** < 0.001**
First delivery operative vaginal delivery	455 (20.1%)	132 (22.6%)	0.18
Induction of labor	697 (30.8%)	208 (35.6%)	**0.02**
Epidural	1,727 (76.2%)	452 (77.4%)	0.56

Data are presented as mean ± standard deviation, median (interquartile range), or *n* (%), as appropriate. *p *values derived from t test or Mann–Whitney U test for continuous variables and the χ^2^ test for categorical variables. Sample size may vary due to missing data

*BMI* body mass index, *EFW* estimated fetal weight

^a^Hypertensive disorders defined as chronic hypertension, preeclampsia, or eclampsia

^b^Diabetes defined as pre-gestational or gestational diabetes

^c^Defined as the difference between sonographic EFW in the index pregnancy and birthweight of the prior delivery

Values shown in bold indicate statistically significant differences between groups (*p* < 0.05)

Intrapartum CD occurred in 68/2,265 patients (3.0%) in the similar-weight group, and 31/584 patients (5.3%) in the higher-weight group (*p* = 0.006). Rates of OVD and maternal and neonatal complications did not differ between groups. Mean birthweight in the index pregnancy was slightly higher in the higher-weight group (3,394 ± 373 g vs 3,355 ± 399 g, *p* = 0.02) (Table 2).

**Table 2:** Delivery outcomes by change between estimated fetal weight and prior delivery birthweight

Variable	Similar-weight group (*n* = 2,265)	Higher-weight group (*n* = 584)	*p value*
Cesarean delivery	68 (3%)	31 (5.3%)	**0.006**
Operative vaginal delivery	57 (2.5%)	19 (3.3%)	0.32
Actual birthweight (g)	3,355 ± 399	3,394 ± 373	**0.02**
PPH	78 (3.4%)	25 (4.3%)	0.33
3rd/4th degree perineal tear	13 (0.6%)	4 (0.7%)	0.95
Shoulder dystocia	13 (0.6%)	7 (1.2%)	0.16

Data are presented as mean ± standard deviation or *n* (%). *p* values derived from t test for continuous variables and χ^2^ or Fisher’s exact tests for categorical variables

*PPH* postpartum hemorrhage

Values shown in bold indicate statistically significant differences between groups (*p* < 0.05)

Indications for intrapartum CD did not differ significantly between groups. Non-reassuring fetal heart rate tracing was the most common indication (72% vs. 68%, *p* = 0.81), followed by arrest disorders (arrest of dilatation or descent; 10.3% vs. 19.3%, *p* = 0.33) in the similar- and higher-weight groups, respectively (Table S1).

In a multivariable logistic regression adjusting for gestational age at delivery, diabetes, induction of labor, maternal BMI, height, and prior OVD, the weight difference category was not independently associated with intrapartum CD (aOR 1.43, 95% CI 0.91–2.27, *p* = 0.12) or OVD (aOR 1.13, 95% CI 0.65–1.96, *p* = 0.68) (Table 3).

**Table 3:** Adjusted multivariable logistic regression comparing mode of delivery by change between estimated fetal weight and prior delivery birthweight

variable	Similar-weight group (*n* = 2,265)	Higher-weight group (*n* = 584)	*p *value
Cesarean delivery aOR	Reference	1.43 (0.91–2.27)	0.12
Operative vaginal delivery aOR	Reference	1.13 (0.65–1.96)	0.68

The similar-weight group served as the reference category. Adjusted odds ratios (aOR) were calculated using multivariable logistic regression models adjusted for gestational age, diabetes, induction of labor, BMI, height, and previous operative vaginal delivery

Induction of labor and prior OVD were independently associated with intrapartum CD. Prior OVD was also independently associated with recurrent OVD, whereas other covariates were not (Table 4).

**Table 4:** Multivariable logistic regression models for factors independently associated with cesarean and operative vaginal delivery in the entire cohort

variable	Cesarean delivery		Operative vaginal delivery	
	aOR (95% CI)	*p value*	aOR (95% CI)	*p value*
Pregnancy age (weeks)	1.21 (0.96–1.52)	0.11	1.11 (0.86–1.43)	0.41
Diabetes^a^	1.19 (0.69–2.04)	0.53	0.85 (0.43–1.68)	0.63
Induction of labor	1.76 (1.16–2.67)	**0.008**	1.8 (1.12–2.9)	**0.01**
BMI	1.04 (1.0–1.08)	0.06	0.99 (0.94–1.03)	0.56
Height (cm)	0.96 (0.92–0.99)	**0.02**	0.97 (0.93–1.01)	0.15
US-EFW (per 100 g)	1.07 (0.99–1.17)	0.07	1.03 (0.95–1.11)	0.52
Previous operative vaginal delivery	2.37 (1.55–3.61)	** < 0.001**	4.17 (2.63–6.63)	** < 0.001**

Adjusted odds ratios (OR) with 95% confidence intervals (CI) were derived from multivariable logistic regression analyses of the entire study population

*BMI* body mass index, *US-EFW* ultrasound estimated fetal weight

^a^Diabetes defined as pre-gestational or gestational diabetes

Values shown in bold indicate statistically significant differences between groups (*p* < 0.05)

Sensitivity analyses incorporating prior birthweight as an additional covariate and temporal analyses adjusting for calendar year yielded materially unchanged results compared with the primary multivariable model. An additional sensitivity analysis including patients with an EFW > 15% lower than previous birthweight within the reference category yielded materially unchanged results (Table S2).

Additional missing-data sensitivity analyses demonstrated stable convergence of the multiple imputation procedure. The mean imputed BMI ranged from 24.81 to 25.52 kg/m^2^, while the adjusted odds ratio for the higher-weight group ranged from 1.39 to 1.52 across the 20 imputations. Complete-case and MNAR sensitivity analyses yielded materially unchanged estimates for the primary association (Table S2).

In subgroup analysis stratified by absolute EFW, no significant association between relative weight increase and intrapartum CD was observed among patients with EFW < 3,500 g (aOR 1.09, 95% CI 0.50–2.41, *p* = 0.82). Among patients with EFW ≥ 3,500 g, the higher-weight group demonstrated higher crude intrapartum CD rates compared with the similar-weight group (7.2% vs 4.1%). However, this association attenuated after multivariable adjustment and was no longer statistically significant (aOR 1.68, 95% CI 0.94–2.99, *P* = 0.08) (Table 5).

**Table 5:** Intrapartum cesarean delivery according to absolute estimated fetal weight strata

EFW (g)	Similar-weight group, *n*	Similar-weight CD, *n* (%)	Higher-weight group, *n*	Higher-weight CD, *n* (%)	Crude OR (95% CI)	Adjusted OR (95% CI)	p value
< 3,500 g	1,532	38 (2.5%)	266	8 (3.0%)	1.22 (0.56–2.64)	1.09 (0.5–2.41)	0.82
≥ 3,500 g	733	30 (4.1%)	318	23 (7.2%)	1.83 (1.04–3.2)	1.68 (0.94–2.99)	0.08

Adjusted odds ratios (aOR) represent the association between increased relative fetal size (higher vs similar-weight group) and intrapartum CD within each EFW subgroup. Adjusted OR were derived from multivariable logistic regression models adjusted for gestational age at delivery, diabetes, induction of labor, BMI, maternal height, and previous operative vaginal delivery

Subgroup analysis stratified according to labor onset demonstrated no independent association between relative fetal weight increase and intrapartum CD among either spontaneous or induced labor (Table S3).

## Discussion

In this retrospective cohort of pregnant patients with a prior vaginal delivery, a clinically meaningful interpregnancy increase in ultrasonographic EFW was not independently associated with intrapartum CD or OVD after adjustment for maternal and obstetric characteristics. The likelihood of achieving successful vaginal delivery remained comparable across groups, even when the EFW in the index pregnancy was substantially higher than the birthweight of the previous neonate.

These findings reinforce the concept that a prior vaginal delivery remains a strong predictor of subsequent delivery success, even in the setting of a larger estimated fetal size, and may help contextualize clinical concerns regarding a “proven pelvis”.

Interestingly, stratification according to absolute EFW demonstrated that no association between relative weight increase and intrapartum CD was observed among patients with EFW < 3500 g. In contrast, among patients with larger estimated fetuses (EFW ≥ 3500 g), the higher-weight group demonstrated increased CD rates, although this association attenuated after adjustment for confounders and did not reach statistical significance. However, these findings suggest a possible trend toward increased intrapartum CD risk among patients with larger absolute EFW, which may warrant further investigation to explore the potential combined effect of relative fetal weight increase and larger absolute fetal size.

Although induction of labor was more common in the higher-weight group, subgroup analyses stratified by labor onset yielded similar results among spontaneous and induced labors, with no independent association between relative fetal weight increase and intrapartum CD observed in either subgroup.

Importantly, the distribution of indications for intrapartum CD did not differ between groups, with non-reassuring fetal heart rate tracing being the most common indication. This finding suggests that intrapartum CD in this cohort likely reflects fetal rather than maternal considerations.

Within this context, the association observed between higher absolute EFW and intrapartum CD in the stratified analysis is consistent with prior reports demonstrating a gradual increase in CD risk with increasing fetal size, even below the macrosomia thresholds [11, 19]. At the same time, the absence of an independent association between relative increases in fetal size and delivery mode in the overall cohort indicates that interpregnancy differences in EFW, when considered alone, may have limited impact on intrapartum outcomes among patients with a prior vaginal delivery.

Consistent with prior literature, a history of OVD was a strong predictor of both intrapartum CD and recurrent OVD in the subsequent pregnancy [20, 21]. In contrast, a substantial increase in EFW relative to previous birthweight was not independently associated with either outcome.

Previous studies evaluating the impact of interpregnancy changes in fetal size on labor outcomes have focused primarily on patients undergoing TOLAC, with inconsistent results [12–15]. In addition, prior research has demonstrated that the clinicians’ perception of fetal size, rather than actual birthweight, may influence intrapartum decision-making and increase the likelihood of CD [11, 19, 22]. However, data addressing this question among patients with a prior vaginal delivery remain limited, underscoring the novelty and clinical relevance of the present study.

Kawakita et al. [6] evaluated whether a current EFW exceeding the birthweight of a prior neonate was associated with intrapartum CD and reported a higher risk when the absolute difference exceeded 500 g. While that study provides valuable insight, our findings differ in several important respects.

First, our cohort was substantially larger. Second, we defined exposure using a relative percentage change rather than an absolute weight difference, allowing each patient to serve as her own reference. Third, we relied exclusively on ultrasound-derived EFW, reflecting contemporary obstetric practice. This approach is supported by evidence demonstrating greater accuracy compared with clinical estimation, particularly in patients with obesity, tall stature, or suspected fetal growth abnormalities [23–26]. Finally, we selected a 15% threshold based on the upper limit of random error reported for commonly used ultrasound formulas, including the Hadlock formula used at our institution, thereby representing a clinically meaningful deviation [17, 18].

From a clinical perspective, these findings suggest that a relatively larger EFW compared with a prior birthweight should not, in isolation, prompt intervention. The results may help inform shared decision-making when counseling patients who express concern that the current fetus appears substantially larger than in previous delivery. Overall, these findings may provide reassurance and support expectant management in the absence of additional risk factors.

### Strengths and limitations

This study has several notable strengths. It is based on a large, well-defined cohort of multiparous patients with two consecutive deliveries, allowing each participant to serve as her own reference. This within-subject design minimizes confounding by nonmodifiable characteristics, such as pelvic anatomy, height, or genetic factors, and helps isolate the influence of other variables that may change between pregnancies.

Restricting the analysis to pregnancies with an EFW between 2,500 and 4,000 g ensured a relatively homogeneous population while excluding extremes of fetal size. This approach reduces management-related bias associated with conditions that independently increase the likelihood of CD, such as fetal growth restriction or macrosomia. Finally, exclusive reliance on sonographic-derived EFW reflects real-world clinical decision-making.

Several limitations should also be acknowledged. First, the retrospective design introduces the potential for selection and residual bias and precludes causal inference. Although we adjusted for key maternal and obstetric variables, unmeasured factors may have influenced outcomes. In addition, residual confounding by management decisions cannot be excluded, as clinicians were aware of the EFW and may have modified intrapartum management, including decisions regarding labor induction or thresholds for intervention, despite the exclusion of planned CD. To reduce potential bias related to fetal size extremes, the analysis was restricted to pregnancies with prior birthweight and antepartum sonographic EFW between 2,500 and 4,000 g.

Second, the exact interval between sonographic EFW assessment and delivery was not individually recorded. However, institutional protocol required that assessments older than 14 days be repeated prior to delivery planning, and a high overall EFW accuracy was observed (median absolute percentage error 5.5%, comparable between study groups), providing indirect support for appropriate timing.

Third, BMI and maternal height data were missing in a considerable proportion of patients, particularly BMI (45.5% and 19.3%, respectively). However, missing data were addressed using multiple imputation. Although the missing-at-random assumption cannot be formally verified, complete-case analyses, evaluation of BMI missingness patterns, and additional MNAR sensitivity analyses all yielded materially unchanged estimates for the primary association, supporting the robustness of our findings. Nevertheless, residual bias due to missing-not-at-random mechanisms cannot be completely excluded.

Fourth, the study period spanned more than a decade, during which temporal changes in obstetric practice may have occurred. However, temporal sensitivity analyses incorporating calendar year yielded unchanged results.

Finally, this was a single-center study conducted at a university-affiliated medical center, which may limit the generalizability of the findings to other clinical settings or populations.

## Conclusions

Among multiparous patients with a prior vaginal delivery, a relative increase in EFW compared with the previous birthweight was not independently associated with a higher risk of intrapartum CD or OVD. A non-significant trend toward increased intrapartum CD risk was observed among patients with larger absolute estimated fetal size; this observation should be interpreted cautiously and requires validation in future investigation. These findings suggest that a clinically meaningful interpregnancy increase in EFW should not, in isolation, alter obstetric management in otherwise uncomplicated pregnancies.

## Supplementary Information

Below is the link to the electronic supplementary material.

Supplementary file1 (DOCX 14 KB)

## Data Availability

No datasets were generated or analyzed during the current study.
