# Supplementary material for: Does a larger estimated fetus compared with prior birthweight increase the risk of cesarean delivery after a prior vaginal birth?
Source: Arch Gynecol Obstet. 2026 Jul 14;313(1):287. doi: 10.1007/s00404-026-08529-6 (PMC13645894; doi:10.1007/s00404-026-08529-6)
Supplement: Supplementary file 1 [file 404_2026_8529_MOESM1_ESM.docx]

**Table S1** Indications for cesarean delivery in the similar- and higher-weight groups

| Cesarean delivery indication | Similar-weight group (n=2,265) | Higher-weight group (n=584) | *p-value* |
| --- | --- | --- | --- |
| NRFHR | 49 (72%) | 21 (67.7%) | 0.81 |
| Arrest disorders | 7 (10.3%) | 6 (19.3%) | 0.33 |

Comparison performed using Fisher’s exact test.
NRFHR - non-reassuring fetal heart rate; Arrest disorders include arrest of dilatation and arrest of descent.

**Table S2** Sensitivity analyses evaluating the robustness of the primary multivariable model under alternative model specifications and missing-data assumptions

| Sensitivity analysis | aOR for intrapartum CD^a^ | 95% CI | *p-value* |
| --- | --- | --- | --- |
| Alternative model specifications | | | |
| Primary model (multiple imputaion) | 1.43 | 0.91-2.27 | 0.12 |
| Complete-case analysis | 1.42 | 0.78-2.6 | 0.25 |
| Inclusion of lower-weight group in reference category | 1.47 | 0.93-2.33 | 0.1 |
| Adjustment for prior birthweight (per 100 g) | 1.13 | 0.55-2.3 | 0.74 |
| Adjustment for calendar year | 1.42 | 0.89-2.24 | 0.14 |
| Alternative missing-data assumptions^b^ | | | |
| MNAR scenario: BMI +2 kg/m^2^ | 1.44 | 0.91-2.28 | 0.12 |
| MNAR scenario: BMI +4 kg/m^2^ | 1.44 | 0.91-2.28 | 0.12 |
| MNAR scenario: BMI +6 kg/m^2^ | 1.44 | 0.91-2.28 | 0.12 |
| MNAR scenario: BMI -2 kg/m^2^ | 1.43 | 0.91-2.27 | 0.12 |

*aOR – adjusted odds ratio; BMI – body mass index; CD – cesarean delivery; CI – confidence interval; MNAR – missing not at random*

^a^ Adjusted odds ratios represent the association between the higher-weight group and intrapartum CD, with the similar-weight group serving as the reference category. For the sensitivity analysis entitled “Inclusion of lower-weight group in reference category”, patients with an estimated fetal weight >15% lower than the previous birthweight were incorporated into the reference group. Models were adjusted for gestational age at delivery, diabetes, induction of labor, sonographic estimated fetal weight, maternal BMI, maternal height, and previous operative vaginal delivery

^b^ Sensitivity analyses were performed under alternative missing-not-at-random (MNAR) assumptions, in which imputed BMI values among women with missing BMI were systematically shifted by +2, +4, +6, and -2 kg/m^2^, while observed BMI values remained unchanged.

**Table S3** Intrapartum cesarean delivery according to labor onset

| Labor onset | Similar-weight group, n | Similar-weight CD, n (%) | Higher-weight group, n | Higher-weight CD, n (%) | Crude OR (95% CI) | aOR (95% CI) | p-value |
| --- | --- | --- | --- | --- | --- | --- | --- |
| spontaneous | 1,568 | 37 (2.4%) | 376 | 15 (4.0%) | 1.72 (0.93-3.17) | 1.4 (0.74-2.65) | 0.3 |
| Induction | 697 | 31 (4.4%) | 208 | 16 (7.7%) | 1.79 (0.96-3.34) | 1.48 (0.76-2.9) | 0.25 |

Adjusted odds ratios (aOR) were derived from multivariable logistic regression models adjusted for gestational age at delivery, diabetes, sonographic EFW, BMI, maternal height, and previous operative vaginal delivery. The similar-weight group served as the reference category.
